# B Vitamin Deficiencies and Associated Neuropathies

**DOI:** 10.1007/s13668-025-00723-3

**Published:** 2026-01-29

**Authors:** Mauricio Alvarez, Santiago Poveda, Alejandra Cisneros, Diana Parra, Maria Luna, Oswaldo Rincón, Issac Guzman

**Affiliations:** 1 Endocrinology Department, Hospital Militar Central, Transversal 3 C #49-02, Bogotá, Colombia; 2 Neurology Program, Universidad Militar Nueva Granada, Bogotá, Colombia; 3 Endocrinology Program, Universidad Militar Nueva Granada, Bogotá, Colombia

**Keywords:** Vitamin B 12, Pyridoxine, Thiamine deficiency, Wernicke encephalopathy, Beriberi

## Abstract

**Purpose of Review:**

This review examines both historical and recent evidence to clarify the current understanding of the relationship between B vitamin deficiencies and neuropathies.

**Recent Findings:**

Vitamin B1 (thiamine) deficiency can lead to neurological disorders such as beriberi and Wernicke’s encephalopathy, conditions with significant prevalence and mortality rates. Vitamin B12 (Cobalamin) is crucial for DNA synthesis, fatty acid metabolism, and myelin production, with its deficiency leading to neuropathies and cognitive disorders. Excess vitamin B6 (pyridoxine), rather than deficiency, appears to be associated with neuropathy.

**Takeaways/Conclusions:**

Vitamin B1 and B12 deficiencies are linked to classic neuropathies, while the connection between vitamin B6 deficiency and neuropathy is less clear, though excess B6 is associated with neurotoxicity. Nutritional deficiencies are less common in developed countries but remain significant in developing nations. In developed countries, factors like alcohol consumption, bariatric surgery, and metformin use are increasing these deficiencies in clinical practice.

## Introduction

Nutritional neuropathies represent a significant global issue, influenced by cultural, geopolitical, and socioeconomic factors. The neurotropic B vitamins -B1, B6, and B12- are critical for neurodevelopment and the proper functioning of the nervous system. Vitamin B1 (Thiamine) deficiency was identified as the cause of Beriberi as early as the seventeenth century, with Carl Wernicke later describing Wernicke-Korsakoff encephalopathy in 1881 [1]. The association between Vitamin B12 (Cobalamin) deficiency and neuropathy became evident in the 1980 s, particularly in cases of subacute combined degeneration of the spinal cord linked to nitrous oxide anesthesia [2].

Vitamin B1 is essential for energy metabolism in neurons and the proper functioning of neurotransmitters; a deficiency can result in neurological disorders such as beriberi and Wernicke’s encephalopathy. Vitamin B6 (Pyridoxine) is vital for neurotransmitter synthesis, including serotonin, dopamine, and GABA, and is involved in amino acid metabolism and myelin production, which is critical for nerve conduction. Vitamin B12 is crucial for DNA synthesis, fatty acid metabolism, and myelin production. This review explores both historical and recent evidence to clarify the current understanding of the relationship between B vitamins deficiency and neuropathies [3, 4].

### Vitamin B1. (Thiamine)

Thiamine, the first member of the water-soluble B vitamin family, functions as a coenzyme in various metabolic reactions. It is essential for multiple cellular functions, including energy metabolism, carbohydrate breakdown, immune system activation, signaling processes, and neuronal communication pathways [3]**.**

Globally, thiamine deficiency is primarily due to poor diets. In developed countries, alcoholic consumption and individuals with chronic diseases are the most affected. Other at-risk groups include pregnant women, dependent elderly individuals, people who have undergone bariatric surgery, individuals with malnutrition, and those who use diuretics chronically [3]**.**

### Sources and Metabolism

Humans require dietary thiamine [4], which is found in foods such as meats, fruits, vegetables, legumes, and cereals. Thiamine deficiency may be more common than expected because cooking and industrial food processing can cause the loss and denaturation of the molecule due to high pH and temperatures [5]**.** The recommended daily intake for a healthy person is 0.5 mg per 1000 kcal.

### Absorption of Thiamine

Thiamine absorption occurs in the duodenum and proximal jejunum and is mediated by saturable co-transporters expressed in the small and large intestines, specifically the human thiamin transporter (hTHTR-1 and hTHTR-2) [6]**.**

Once absorbed, thiamine passes through the intestinal mucosal cells and enters the bloodstream. Approximately 80% is then captured by erythrocytes and retained through phosphorylation, converting it to its active form [7]**.** Thiamine reaches the central nervous system after crossing the blood-brain barrier, distributing differently across various parts of the CNS. In cases of thiamine deficiency, the most affected brain regions are the cerebellum, mammillary bodies, thalamus, hypothalamus, and brainstem [8]**.**

### Functions

Thiamine functions as a coenzyme for enzymes primarily located in mitochondria, playing a crucial role in cellular metabolism by acting as a cofactor for three essential enzymes: transketolase (TKT), pyruvate dehydrogenase (PDH), and alpha-ketoglutarate dehydrogenase (α-KGDH). These enzymes catalyze fundamental biochemical reactions that contribute to various metabolic processes [9].

TKT participates in the pentose phosphate pathway, generating two important molecules: ribose-5-phosphate, a pentose crucial for nucleic acid synthesis, and reduced nicotinamide adenine dinucleotide phosphate (NADPH). NADPH plays a crucial role in reduction reactions, providing hydrogen atoms essential for the synthesis of fatty acids, steroids, amino acids, and certain neurotransmitters. TKT activity is the most affected by variations in thiamine concentrations in brain regions [10].

Consequently, thiamine deficiency is expected to lead to increased oxidative stress and reduced cell proliferation, as well as decreased synthesis of fatty acids, including myelin, with severe consequences, especially during neurogenesis in the cortex and hippocampus [11].

The enzymes pyruvate dehydrogenase and alpha-ketoglutarate dehydrogenase are involved in the Krebs cycle, a central metabolic pathway for ATP generation. Therefore, a decrease in their activity leads to cellular damage and even neuronal cell death [12].

### Pathophysiology of Thiamine Deficiency

Prolonged thiamine deficiency affects both the central and peripheral nervous systems and can result from insufficient nutrient intake, impaired absorption, increased requirements, or increased losses [13].

The most common cause in developed countries is alcoholism, though other patient groups are also at risk of developing the deficiency. These include malnourished individuals, those infected with HIV, patients undergoing chemotherapy, and those with impaired thiamine absorption [13].

One of the most well-known mechanisms of thiamine deficiency is associated with chronic alcohol consumption. This deficiency primarily arises from inadequate dietary intake during alcohol use. Additionally, the presence of alcohol reduces hepatic storage and gastrointestinal absorption of thiamine due to decreased expression of THTR, which is essential for intestinal absorption. Moreover, alcohol decreases thiamine phosphorylation, thereby impairing its subsequent cellular utilization. Additionally, patients with diabetes exhibit increased thiamine clearance due to the downregulation of thiamine transporters THTR-1 and THTR-2, resulting in thiamine deficiency [14, 15].

Thiamine deficiency leads to functional changes in neurotransmission, most notably within the glutamatergic and GABAergic systems, resulting in a toxic neuroexcitatory state. Disruptions in the pentose phosphate pathway lead to decreased neuronal myelination and subsequent alterations in signaling [15].

.

### Clinical Manifestations

Thiamine deficiency in the diet leads to two primary clinical phenotypes, affecting both the central and peripheral nervous systems:

### Dry Beriberi

Characterized by a symmetric mixed polyneuropathy, presenting with paresthesia, muscle weakness, and alterations in deep tendon reflexes due to demyelination of peripheral nerve fibers, with no acute inflammatory reaction observed histologically [13, 15]**.**

### Wernicke-Korsakoff Syndrome

Wernicke’s encephalopathy(WE) and Korsakoff syndrome are among the most severe central nervous system manifestations of thiamine deficiency. These conditions are explained by apoptotic cell death due to N-methyl-D-aspartate (NMDA) toxicity. Autopsy studies have shown a prevalence of 12.5% for Wernicke’s encephalopathy among individuals with chronic alcohol use, with a higher prevalence in men; however, women are more sensitive to developing this condition [16, 17].

The clinical difference between these two conditions is that WE is an acute syndrome with high morbidity and mortality, while Korsakoff syndrome refers to a chronic neurologic condition that typically occurs as a consequence of WE [17, 18].

**Fig. 1:**
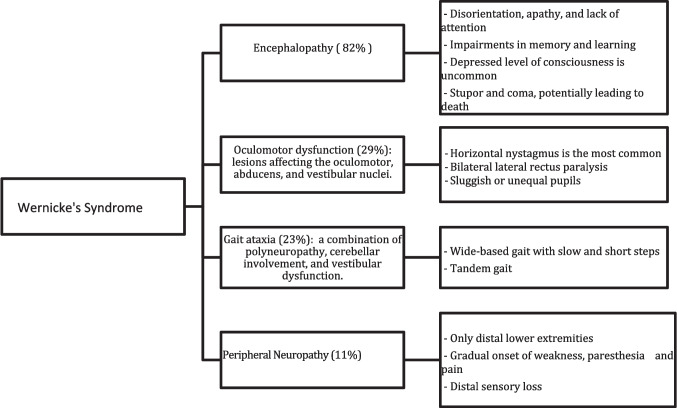
Wernicke’s Syndrome manifestations and signs

The classic manifestations of the acute clinical presentation include the triad of encephalopathy, oculomotor dysfunction, and gait ataxia. This triad is present in only one-third of patients, with ataxia often being the earliest symptom [17]. Figure 1.

Diagnosis of WE using two out of four Caine criteria yields a sensitivity of 85% and a specificity of 44% [18, 19]**.**

Korsakoff syndrome is diagnosed mainly through clinical presentation, with severe anterograde amnesia being a key feature. It lacks standardized diagnostic criteria, and diagnosis relies on identifying significant memory impairments and excluding other causes of cognitive deficits [18, 19]**.**

### Laboratory Tests

The most reliable assessment of thiamine deficiency is the evaluation of erythrocyte transketolase activity (ETKA) in vitro by measuring its activity before and after thiamine administration. A low ETKA with an increase of more than 25% after thiamine supplementation confirms the diagnosis of thiamine deficiency [18, 19]**.**

It is also possible to measure thiamine and its metabolites in plasma and urine; however, these tests are less reliable. They reflect recent thiamine intake rather than the overall thiamine status and are often not readily available [20–22]**.**

### Imaging

Computed tomography (CT) has low sensitive for detecting thiamine deficiency-related lesions. Magnetic resonance imaging (MRI) is more effective for identifying acute diencephalic and periventricular lesions. Typical MRI findings include areas of increased signal on T2-weighted and FLAIR images, decreased signal on T1-weighted images, and diffusion abnormalities around the aqueduct and third ventricle, as well as within the medial thalamus, dorsal medulla, tectal plate, and mammillary bodies. Lesions may also be observed in regions such as the cerebellum and cranial nerve nuclei [21]**.**

### Treatment

A recommended regimen involves administering 500 mg of thiamine intravenously over 30 minutes three times daily for two consecutive days, followed by 250 mg IV or IM once daily for an additional five days. Administering glucose without prior thiamine can exacerbate Wernicke**’**s encephalopathy (WE); therefore, thiamine should be given before glucose [23, 24]**.**

Due to erratic gastrointestinal absorption of thiamine in malnourished patients and those with a history of chronic alcohol use, oral thiamine administration is an unreliable initial treatment for WE [25]**.**

### Differential Diagnosis

Thiamine deficiency has a broad differential diagnosis that includes psychiatric disorders and organic diseases. A detailed medical history, including dietary habits, alcohol consumption, and medication use, is essential for guiding the diagnosis [26]**.**

Physical examination, with a focus on the nervous and cardiovascular systems, helps identify signs consistent with peripheral neuropathy, cardiomyopathy, or autonomic decompensation. Complementary studies, such as laboratory tests and neuroimaging, are necessary to confirm the diagnosis and exclude other conditions [26]**.**

### Vitamin B6 (Pyridoxine)

Vitamin B6 is a water-soluble vitamin that encompasses three pyridine derivatives: pyridoxine, pyridoxal, and pyridoxamine. Pyridoxine is the form most commonly found in dietary supplements. Through phosphorylation mediated by pyridoxal kinase, either alone or in combination with pyridoxine 5′-phosphate oxidase, vitamin B6 is converted into its active form, a phosphate ester derivative known as pyridoxal 5′-phosphate (PLP). This bioactive coenzyme form serves as an essential cofactor in the metabolism of glucose, lipids, and amino acids, as well as in neurotransmitter synthesis [27–30].

Humans cannot synthesize vitamin B6, so it must be obtained through the diet. Its absorption occurs primarily in the small intestine via passive diffusion. The recommended daily intake of vitamin B6 ranges between 1.5 and 1.8 mg per day. Due to its abundance in food sources, vitamin B6 deficiency is rare [31]. Once absorbed, vitamin B6 is transported to the liver, where it undergoes phosphorylation to become its active form. PLP is then distributed to various tissues in the body, including the brain and nerves, where it participates in a range of PLP-dependent enzymatic reactions [32, 33].

The biological reactions catalyzed by PLP-dependent enzymes are crucial for several essential processes, including the biosynthesis of hemoglobin and amino acids, as well as the metabolism of fatty acids and carbohydrates [34]. In the brain, PLP serves as a key cofactor for the enzyme L-aromatic amino acid decarboxylase, which catalyzes the synthesis of two major neurotransmitters: serotonin from the amino acid tryptophan and dopamine from L-3,4-dihydroxyphenylalanine (L-DOPA). Additionally, PLP is a cofactor for glutamate decarboxylase, which catalyzes the synthesis of gamma-aminobutyric acid (GABA) from glutamate. Other neurotransmitters, including glycine, D-serine, and histamine, are also synthesized in reactions catalyzed by PLP-dependent enzymes. Corresponding to its role in the aforementioned neurotransmitters, pyridoxine influences adrenergic, serotonergic, and glutamatergic systems, thereby impacting normal nerve signal transmission [35, 36].

Additionally, PLP acts as a cofactor for two crucial enzymes in homocysteine metabolism: cystathionine beta-synthase and cystathionase. Therefore, vitamin B6 deficiency results in elevated plasma homocysteine levels, leading to neurotoxicity, promoting oxidative stress, and inducing neuronal apoptosis [34, 36]. PLP is also essential as a cofactor in the synthesis of sphingolipids, which are critical for myelin formation. Myelin, a protective sheath around nerve fibers, enables the rapid and efficient transmission of electrical impulses. Vitamin B6 deficiency can lead to demyelination, resulting in impaired nerve transmission [36, 37]. Due to its significant role as a coenzyme in pathways responsible for neurotransmitter and myelin synthesis, vitamin B6 deficiency can severely impair both the central nervous system (CNS) and the peripheral nervous system (PNS).

### Vitamin B6 Deficiency and Neuropathies

Vitamin B6 deficiency is associated with anemia, seborrheic dermatitis, glossitis, stomatitis, microcytic anemia, and weakened immune function. Neurological symptoms range from cognitive impairment, seizures, and depression—primarily affecting the central nervous system (CNS)—to carpal tunnel syndrome and peripheral neuropathy mainly sensitive, which manifest as paresthesia, painful dysesthesia, and thermal sensory disturbances in the peripheral nervous system (PNS) [36–38].

Two clinical syndromes are described based on the age of onset. In infants from weeks to a few months old, vitamin B6 deficiency is characterized by irritability, abnormal acute hearing, and recurrent seizures that are abrupt and difficult to control with conventional antiseizure drugs. These cases are typically due to dietary vitamin B6 deficiency, commonly from breastfeeding by malnourished mothers. The patients usually have a normal birth history and remain healthy until the onset of symptoms, which cease once the dietary deficiency is corrected, allowing for normal development. Another rare form of pyridoxine-sensitive seizures occurs in infants with congenital errors in PLP metabolism, caused by mutations in genes such as ALDH7A1, ALDH4A1, PNPO, TNSALP, PLPBP, and PLPHP. In these cases, seizures appear in the neonatal period, respond poorly to antiseizure drugs, and require prolonged administration of high doses of pyridoxine or PLP for control and to minimize adverse effects on cognitive development [38].

Although adults are more tolerant of vitamin B6 deficiency, there is a high prevalence of deficiency in pregnant or lactating women, the elderly, individuals predisposed to malabsorption (e.g., those with a history of bariatric surgery or small intestine diseases), and patients with renal insufficiency undergoing hemodialysis. Medications such as isoniazid, hydralazine, penicillamine, and levodopa/carbidopa intestinal gel have also been associated with pyridoxine deficiency. Additionally, excessive alcohol consumption and its metabolite, acetaldehyde, lead to the degradation of PLP [32, 39].

Most of the evidence linking vitamin B6 deficiency to neuropathy is based on studies reporting neuropathy associated with isoniazid, hydralazine, and levodopa/carbidopa. Chronic vitamin B6 deficiency is associated with an axonal disease characterized by a length-dependent pattern subacute sensory or sensorimotor neuropathy, with paresthesia and ataxia being the main features, although the presence of pain, which can be disabling, has also been reported. Motor weakness is generally minimal, and optic neuropathy has also been documented. Sensory symptoms typically first appear in the feet. The neurological examination is characterized by altered sensation and weakness in a distal-to-proximal pattern, along with decreased or absent deep tendon reflexes [32, 39].

.However, a meta-analysis evaluating the role of vitamin B6 in peripheral neuropathy, including six studies, revealed that although low vitamin B6 levels are observed in patients with peripheral neuropathy of various etiologies, this finding is generally associated with an overall poor nutritional status, including low levels of other essential vitamins such as vitamin B12, whose deficiency can also cause peripheral neuropathy. Additionally, low vitamin B6 levels may result from adverse effects of treatments for conditions that predispose individuals to neuropathy, such as diabetes and chronic renal insufficiency.

Therefore, there is no conclusive evidence of a direct causal relationship between vitamin B6 deficiency and peripheral neuropathy [32]. A recent systematic review found that elevated levels of vitamin B6 are associated with axonal sensory neuropathy, suggesting a neurotoxic effect of vitamin B6. Although the physiological function of this vitamin and other studies suggest that deficiency is a potential risk factor for neuropathy, there appears to be more evidence supporting the harmful effects of excess vitamin B6 [40].

Several studies have suggested a subjective improvement in neuropathy symptoms in patients with peripheral neuropathy of various etiologies following the administration of vitamin B6 supplements. However, in none of these studies was vitamin B6 administered as monotherapy; it was always part of a combined treatment with other vitamins. Thus, the potential therapeutic role of vitamin B6 cannot be confirmed with the current data [40].

### Vitamin B12 (Cobalamin)

#### Physiology

Vitamin B12 (B12) consists of a group of organic compounds containing a cobalamin (Cbl) structure and is a water-soluble vitamin predominantly obtained from animal-derived foods. These compounds include adenosylcobalamin (AdoCbl), cyanocobalamin (CNCbl), hydroxycobalamin (HoCbl), and methylcobalamin (MeCbl) [41]. Due to its structure, B12 requires three proteins—intrinsic factor, haptocorrin, and transcobalamin—for its absorption and transport, as well as two enzymatic conversions in the form of cofactors via methionine synthase and methylmalonyl-CoA mutase [42, 43]. Deficiency in B12 leads to hematological, psychiatric, and neurological manifestations [44]. This review will focus on neuropathy associated with B12 deficiency.

Vitamin B12 is exclusively synthesized by bacteria and is present in foods such as eggs, milk, red meat, and poultry products. The human intestinal microbiota cannot produce sufficient vitamin B12 to meet daily needs [45]. There are two methods for B12 absorption: a passive method that accounts for less than 1% of absorption, and an active method in the ileum facilitated by intrinsic factor (IF) [46].

Vitamin B12 (B12) is ingested bound to dietary proteins and initially binds to haptocorrin (HC), which protects it from the acidic gastric environment, forming the B12-HC complex. Upon reaching the small intestine, haptocorrin is hydrolyzed in the duodenum by pancreatic proteases. Free B12 then binds to intrinsic factor (IF) produced by gastric parietal cells, forming the B12-IF complex. This complex is absorbed in the distal ileum through the cubam complex located on the apical surface of enterocytes. The cubam complex is composed of cubilin and amnionless, with additional proteins such as megalin (MAG) and receptor-associated protein (RAP) potentially contributing [47–49].

Inside the enterocyte, the B12-IF complex is transported to the lysosome, while the cubam complex is recycled back to the plasma membrane. In the lysosome, intrinsic factor is degraded, releasing B12, which is then transported to the cytoplasm. Free B12 is actively transported by a multispecific membrane transporter, multidrug-resistant protein 1 (MRP1/ABCC), and exits the cell through the basolateral membrane into the bloodstream [42].

In the blood, approximately 80% of vitamin B12 binds to haptocorrin (HC), forming holohaptocorrin (B12-HC). This complex represents the bioavailable form that can be absorbed by peripheral cells, including those in the nervous system, or transported to the liver to establish hepatic reserves via endocytosis by the asialoglycoprotein receptor (ASGPR). Additionally, B12 from this complex can be transferred to transcobalamin (TC), serving as a circulating reserve. Only a small fraction of B12 circulates freely in the blood [47].

Vitamin B12 is primarily excreted through bile in the enterohepatic circulation, with an estimated 30 to 60% of the daily oral intake being excreted in the feces [46, 50]. The bioavailability of B12 from food is estimated to be 50% in individuals without malabsorption issues and 55 to 74% in crystalline B12 found in fortified foods and supplements [42]. According to the European Food Safety Authority, the recommended daily intake of B12 for adults is 4 μg/day [49], while the US recommends 2.4 μg/day [51]. Blood levels considered normal vary between 150 and 900 pg/ml [46].

Hypovitaminosis B12 is the most extensively studied form of vitamin B12 deficiency and primarily arises from inadequate intake among vegetarians, vegans, the elderly, individuals with malnutrition, alcoholics, those with infections, pregnant women, certain medications, genetic abnormalities affecting B12 receptors in enterocytes, or conditions that compromise intestinal absorption, such as atrophic gastritis. Due to the presence of hepatic B12 reserves, it may take years for symptoms to manifest [50–52].

.

Research suggests that the effects of B12 deficiency extend beyond insufficient intake to include molecular and cellular alterations, such as oxidative stress accumulation and changes in lysosomal activity [47, 53].

**Fig. 2:**
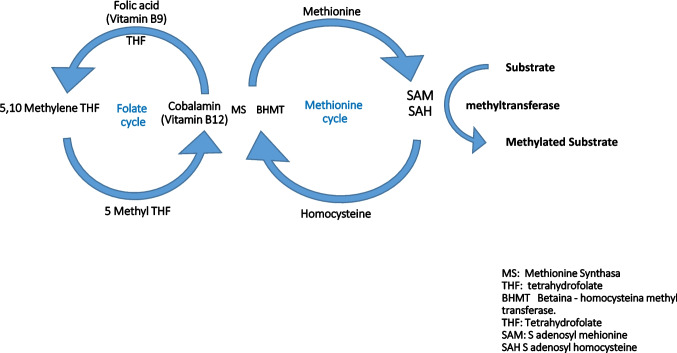
Mehionine and folate Cycles

Vitamin B12, when free in the cytoplasm, can be converted into its active forms, adenosylcobalamin (AdoCbl) and methylcobalamin (MeCbl). These act as cofactors for mitochondrial methylmalonyl-CoA mutase (MCM) and cytosolic methionine synthase (MS), respectively. AdoCbl assists in converting L-methylmalonyl-CoA to succinyl-CoA via MCM, enabling its entry into the Krebs cycle. MeCbl is required by MS to convert homocysteine to methionine (MET) in the sulfur amino acid pathway. MET is essential not only for protein, neurotransmitter, and DNA synthesis but also as a precursor to S-adenosylmethionine, which is crucial for maintaining myelin sheaths (Fig. 2). Consequently, disruptions in these processes can lead to demyelination and impaired DNA repair [36, 54].

### Vitamin B12 Deficiency Related Neuropathies

Vitamin B12 deficiency affects the nervous system through demyelination of both the central and peripheral nervous systems, leading to neuropsychiatric disturbances, optic neuropathy, myelopathy, and peripheral neuropathy [55, 56]. Vitamin B12 is essential for myelin synthesis, nerve metabolism, and neuronal regeneration [36]. The pathogenesis of neuropathies due to B12 deficiency is related to cobalamin’s role in the synthesis and maintenance of the myelin sheath and the accumulation of neurotoxins such as homocysteine and methyl malonic acid, which trigger inflammation, oxidative stress, and microvascular disease [32, 55]. Although cobalamin deficiency impacts myelin formation, the most common finding in neuropathy associated with vitamin B12 deficiency is chronic axonal sensory polyneuropathy [57].

Nutritionally acquired neuropathies require prompt diagnosis and treatment, as early and appropriate intervention can stabilize or reverse the condition. Symptom onset is heterogeneous and often insidious, although compared to idiopathic polyneuropathies, it is more likely to present acutely. Initial symptoms include paresthesia, hypoesthesia, and weakness in the hands or symmetrically in both hands and feet [56, 58]. Weakness and gait instability may develop later. Neuropathic pain is a less prevalent symptom [59].

Neurological symptoms are more frequent and severe when the B12 deficiency is more profound and can be the first and only manifestation of the deficiency. Neuropathy is detected during physical examination in 25% of patients with B12 deficiency. Neurological examination typically reveals signs of peripheral nerve involvement, although it may also be accompanied by signs of posterior spinal cord involvement if the deficit extends to this level (Table 1). If proprioceptive impairment is severe, the Romberg sign may be present. There is no consistent sequence in the appearance of symptoms between peripheral nerve and spinal cord involvement [38].

**Table 1:** Distinguishing Signs Between Neuropathy and Myelopathy in Vitamin B12 Deficiency

Neuropathy	Myelopathy
Glove-and-stocking hypoesthesia Hyporeflexia or areflexia Distal > proximal limb paresis	Weakness Hyperreflexia Spasticity Extensor plantar response Hypopallesthesia Proprioceptive impairment Romberg sign

Neurological sequelae of cobalamin deficiency may take between 2 and 5 years to manifest due to hepatic reserves [38]. In elderly patients, identifying symptoms related to B12 deficiency can be challenging because they may be confused with manifestations of other common age-related diseases [55]. Relapses are generally associated with the same neurological phenotype [60].

### Diagnosis

Diagnostic testing for B12 deficiency should be performed in patients with clinical suspicion of the deficiency, including those with macrocytic anemia or neurological symptoms, as well as those presenting with nonspecific neurological symptoms in the elderly, low B12 intake diets, fertility issues, or gastrointestinal disorders [61]. The measurement of serum vitamin B12 levels may have low specificity for identifying true deficiencies when they are at the lower end of normal (200–300 pg/mL). In these cases, measuring homocysteine and methylmalonic acid, which are cobalamin-dependent precursors, can be useful [55, 62]. However, elevated levels of homocysteine and methylmalonic acid are not specific to cobalamin deficiency (Table 2) [63].

**Table 2:** Factors Elevating Methylmalonic Acid and Homocysteine. Adapted from [63]

Elevated Methylmalonic Acid	Elevated Homocysteine
Vitamin B12 Deficiency Renal Insufficiency Inherited Metabolic Disorders Hypovolemia	Vitamin B12, B9, or B6 Deficiency Renal Insufficiency Hypothyroidism Hypovolemia Psoriasis Inherited Metabolic Disorders

**Fig. 3:**
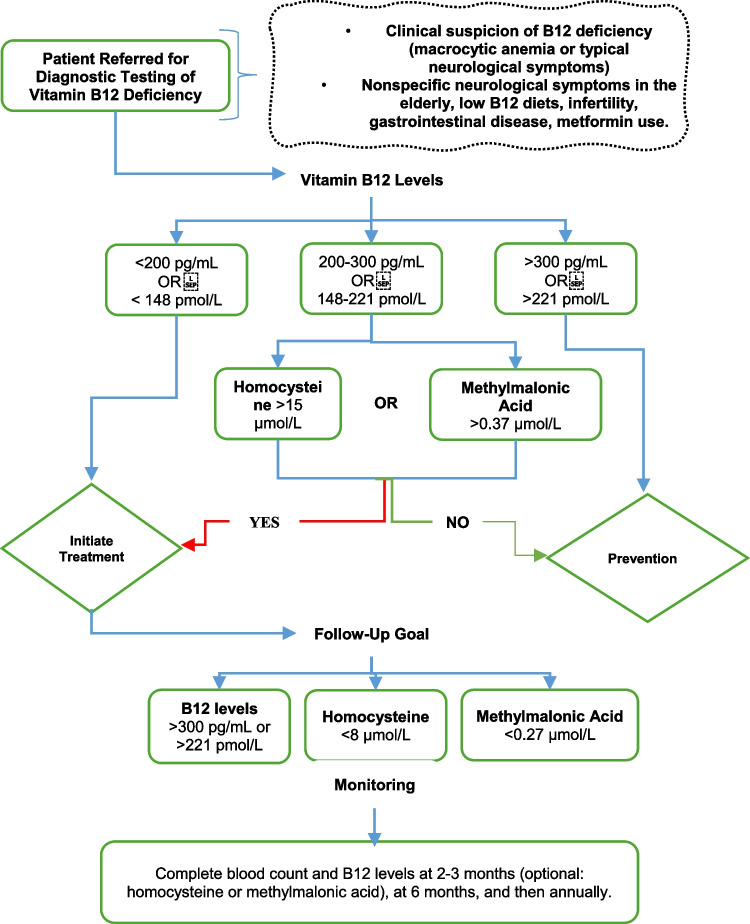
Algorithm for Diagnosis, Treatment, and Follow-up in Vitamin B12 Deficiency. Adapted from [61, 62]

Serum homocysteine levels should be measured in a fasting state, and the sample should be refrigerated immediately to prevent elevation due to ambient temperature [63]. Methylmalonic acid levels are the most sensitive and specific marker for confirming vitamin B12 deficiency and are often used as the gold standard; however, they are less available and expensive [62]. Figure 3.

It is recommended to evaluate serum cobalamin and folate levels together due to the overlap in their metabolic pathways and the possibility of a combined deficiency [64] The most common hematological manifestation is macrocytic anemia, present in up to 50% of patients; however, it may be absent at the time of neurological symptom onset and is therefore insufficiently sensitive for diagnostic purposes [63].

Neurophysiological studies can reveal a range of findings, such as axonal neuropathy, sensory polyneuropathy, and sensory neuronopathy [64], with axonal neuropathy being the most frequent (57–76%), followed by demyelinating neuropathy (5–24%) (Table 3) [31]. Nerve conduction studies show abnormalities in approximately 50–55% of patients with neuropathy symptoms [63, 64].

**Table 3:** Characteristics of Neurophysiological Studies in Axonal and Demyelinating Neuropathy. CMAP: Compound Muscle Action Potential, SNAP: Sensory Nerve Action Potential

Axonal Neuropathy.	Demyelinating Neuropathy.
- Reduction in CMAP - Reduction in SNAP - Normal or slightly slowed conduction velocities	- Decreased conduction velocity - Prolonged distal latencies - Minimal F-wave latencies - Conduction blocks

In less common cases, demyelinating sensory-motor neuropathies or pure motor axonal neuropathies may be observed [57]. Small fiber neuropathy, responsible for neuropathic pain, is characterized by the loss of unmyelinated C fibers and A-delta fibers. Diagnostic tests such as quantitative sensory testing (QST) and quantitative sudomotor axon reflex testing (QSART) are used, with histopathological evidence of small fiber neuropathy in skin biopsy being the gold standard [58, 59]. Pathological examination of the peripheral nervous system reveals acute axonal degeneration in early stages and chronic axonopathy with demyelination in late stages [63, 65].

### Etiology and Differential Diagnosis

To determine the cause of vitamin B12 deficiency, it is essential to establish whether it is due to low intake or gastrointestinal malabsorption.

In evaluating vitamin B12 deficiency, the measurement of intrinsic factor autoantibodies and anti-parietal cell antibodies should be considered to detect pernicious anemia, as well as levels of gastrin and pepsinogen to identify atrophic gastritis. The Schilling test is a historical diagnostic procedure used to assess vitamin B12 absorption and identify the underlying cause of vitamin B12 deficiency. It involved administering radioactive vitamin B12 and subsequently measuring its urinary excretion. Absorption was considered normal if urinary excretion exceeded 8% and impaired if it was below this threshold. The Schilling test is no longer used in clinical practice due to the risks associated with radiation exposure and the availability of alternative diagnostic methods. Upper endoscopy is recommended if pernicious anemia is suspected to confirm atrophic gastritis and exclude other non-immune causes [66].

Differential diagnosis should include a complete blood count, thyroid function tests, HIV testing, measurement of vitamin E, B9, B6, and B1 levels, glucose levels, and autoimmune profiles. Additionally, investigation of medication uses or exposure to toxins such as alcohol and heavy metals is warranted.

Diabetic neuropathy is a common complication in diabetic patients, affecting more than 50% of individuals, and it can present as sensory, motor, and autonomic neuropathy. The etiology of diabetic neuropathy is multifactorial. Vitamin B12 deficiency has been linked in multiple studies to the onset and progression of diabetic neuropathy, and systematic reviews and meta-analyses have demonstrated that vitamin B12 supplementation is crucial in this population, as it can improve symptoms of peripheral neuropathy [67–69].

### Treatment

The treatment of vitamin B12 deficiency depends on the underlying cause; however, in cases with neurological involvement, early initiation of parenteral treatment is preferred. Once symptoms improve, treatment may be switched to oral administration [61]. A transient worsening of neurological symptoms may occur before improvement [65].

Parenteral administration of vitamin B12 is common, although a Cochrane review found that both oral and intramuscular replacement methods are equally effective for normalizing serum B12 levels [66]. The duration of treatment depends on whether the underlying cause of the deficiency persists. Patients with B12 deficiency due to absorption issues require indefinite monthly intramuscular administration [55].

Treatment Regimen:

- Parenteral: Intramuscular cyanocobalamin: 1 mg daily for 1 week, then 1 mg weekly for 1 month, followed by 1 mg monthly.
- Oral: 1–2 mg orally daily.

Monitoring should include a complete blood count and vitamin B12 levels at 2–3 months, then at 6 months, and subsequently annually. Homocysteine or methylmalonic acid levels should normalize within the first 10–14 days of treatment; if they do not improve or if neurological symptoms progress, an alternative diagnosis should be considered [60].

A stepwise diagnostic approach using empirical vitamin B12 supplementation followed by a reticulocyte response test and subsequent folate supplementation, if needed, could be a cost-effective strategy for differentiating megaloblastic anemia due to B12 and folate deficiency. Since B12 deficiency can lead to irreversible neurological damage, initiating a trial of parenteral B12 (e.g., 1000 μg intramuscularly) is a safe and inexpensive first step. A reticulocyte response, typically peaking within 5–7 days, suggests B12 deficiency as the primary cause, whereas a lack of response indicates the need for further evaluation, such as a folate trial. This method is advantageous as it avoids unnecessary initial laboratory testing (e.g., serum B12, folate, MMA, homocysteine), relies on functional testing with a reticulocyte count, prevents neurological complications by addressing B12 deficiency first, and provides a rapid turnaround within one to two weeks. However, it may delay definitive diagnosis in cases of combined deficiencies and is not suitable for patients with severe symptoms, such as profound pancytopenia or neurological involvement, where urgent testing is necessary. Additionally, while this strategy identifies the deficient vitamin, it does not determine the underlying cause, such as pernicious anemia, malabsorption, or dietary deficiency [70].

### Prevention

Promote the consumption of foods rich in vitamin B12 (such as seafood, meat, eggs, and dairy products) and fortified foods (including wheat flour, bread, cereals, nutritional bars, and energy drinks). Routine administration of vitamin B12 supplements is not recommended for individuals without documented deficiency who consume a varied diet.

### Prognosis

Neurological improvement in patients with vitamin B12 deficiency is gradual and may be incomplete, typically occurring between 6 and 12 months after the initiation of therapy; no further improvement is expected after this period. Recovery of neurological symptoms is inversely correlated with the time elapsed between the onset of symptoms and the start of treatment [63]. Studies have shown that 47% of patients experienced complete neurological recovery, while 6% had moderate to severe long-term neurological disability [71]. There is no evidence that higher doses of parenteral vitamin B12 accelerate neurological recovery [63].

With appropriate treatment, improvement in nerve conduction parameters may be observed within six months of therapy [43]. Paresthesia without sensory loss or motor weakness are the symptoms most likely to be fully resolved [55]. In patients who discontinue vitamin B12 supplementation after clinical recovery, neurological symptoms may recur within 6 months [43].

### Vitamin B9 (Folic Acid)

Folate is a water-soluble B vitamin required for the synthesis of pyrimidines and purines, essential for DNA replication and cell mitosis. It plays a crucial role during early embryogenesis in neural growth and development and is vital in preventing congenital disabilities, particularly neural tube defects. Major dietary sources of folate include green leafy vegetables such as broccoli, citrus fruits, and fortified foods. In adults, folate is important for nervous system repair [61, 72–74].

Folate deficiency can result from inadequate dietary intake, impaired absorption, increased requirements, medication use, alcohol consumption, congenital disorders, and secondary to vitamin B12 deficiency. The latter occurs due to impaired methionine synthase activity, leading to the trapping of folate as methyltetrahydrofolate (Fig. 2), a phenomenon known as the folate trap, which results in increased urinary folate loss [72, 73].

### Clinical Manifestations of Folate Related Neuropathy

Patients with folate deficiency often develop peripheral neuropathy, and folate deficiency is considered a risk factor for neuropathy, particularly in individuals younger than 40 years [73]. Folate deficiency neuropathy presents as a predominantly sensory neuropathy rather than motor, affecting deep sensation more than superficial, with slow progression and preservation of biceps tendon reflexes. Compared to thiamine deficiency neuropathy, it progresses more gradually [74].

### Diagnosis of Folate Deficiency

Folate levels can be measured in serum and red blood cells. Serum folate reflects recent dietary intake, whereas red blood cell folate represents tissue folate status over the lifespan of erythrocytes, thus providing an assessment of folate levels over the past few months.

Folate deficiency is defined as serum folate <6.8 nmol/L (3 ng/mL), folate insufficiency as serum folate 6.8 to <13.6 nmol/L, and folate sufficiency as folate ≥13.6 nmol/L (5.9 ng/mL) according to World Health Organization (WHO) guidelines [75]. A serum folate concentration above 25.5 nmol/L (11 ng/mL) is considered optimal for reducing the risk of neural tube defects. In adults, a level above 10 nmol/L (4.4 ng/mL) prevent homocysteine elevation [61, 76, 77].

A red blood cell folate level below 906 nmol/L (400 ng/mL) is considered insufficient for optimal neural tube defect prevention, while a level below 340 nmol/L (149 ng/mL) is classically associated with clinical folate deficiency [77]. However, in most cases, red blood cell folate measurement is not necessary [61].

Elevated homocysteine levels are a sensitive indicator of folate status and correlate strongly with serum folate levels in the lower physiological range, increasing when serum folate falls below 10 nmol/L (4.4 ng/mL). However, this finding is not specific to folate deficiency. In cases of diagnostic uncertainty regarding folate deficiency or in special circumstances, measuring homocysteine levels can be useful. A value greater than 15 μmol/L is indicative of folate deficiency; however, results should be interpreted based on local reference ranges [61].

Electrophysiological studies typically show findings consistent with axonal neuropathy, while pathological examination reveals large-fiber axonal loss without segmental demyelination [74].

### Treatment of Folate Deficiency

Treatment consists of folic acid supplementation, with the dosage depending on the underlying cause of the deficiency. A dose of 0.8 mg/day or higher is required for maximal reduction of homocysteine levels. It is estimated that every 100 mcg/day of folic acid intake increases serum folate concentration by 11% [61, 76].

It is crucial to assess concurrent vitamin B12 deficiency, as inappropriate folic acid supplementation in B12 deficiency may lead to neurological and hematological deterioration [61].

For megaloblastic anemia, a dose of 5 mg/day for four months is recommended, while in pregnancy, supplementation should continue until term. In malabsorption states, doses may be increased to 15 mg/day. For prophylaxis in conditions such as chronic hemolysis or dialysis, doses range from 5 mg daily to weekly, depending on dietary intake and dialysis frequency. In pregnancy, prophylactic doses range from 200 to 500 mcg/day [61].

For neuropathy due to folate deficiency, doses between 800 and 1000 mcg/day may be considered, extrapolated from studies aiming to rapidly normalize red blood cell folate levels [78, 79]. In patients with diabetic polyneuropathy, a study found that folic acid supplementation at 1 mg/day for 16 weeks decreased homocysteine levels without affecting vitamin B12 levels. Additionally, it increased sural nerve sensory amplitude and conduction velocity, suggesting a potential benefit in enhancing nerve conduction in diabetic neuropathy [78].

Folate deficiency has been associated with increased all-cause mortality, as well as cardiovascular disease and cancer-related mortality [80].

## Conclusions

Neurotropic B vitamins, a group within the B-complex vitamins, play a crucial role in the development and function of the nervous system. The key neurotropic B vitamins include: Vitamin B1 (Thiamine), essential for energy metabolism in neurons and the proper functioning of neurotransmitters; a deficiency can result in neurological disorders such as beriberi and Wernicke’s encephalopathy.

Vitamin B6 (Pyridoxine) is vital for neurotransmitter synthesis, including serotonin, dopamine, and GABA, and is involved in amino acid metabolism and myelin production, which is critical for nerve conduction. Recent evidence suggests that excess vitamin B6, rather than deficiency, is more strongly associated with neuropathy. However, from a physiological perspective, it is expected that deficiency would also be a risk factor for neuropathies, as has been suggested in some studies.

Vitamin B12 (Cobalamin) and B9 (folic acid) are crucial for DNA synthesis, fatty acid metabolism, and myelin production, with its deficiency leading to neuropathies, megaloblastic anemia, and cognitive disorders. These vitamins are essential for nervous system health, and their deficiency can have severe consequences on neurological function.

## Data Availability

No datasets were generated or analysed during the current study.
